# Supplementary material for: Detection of Fungi and Oomycetes by Volatiles Using E-Nose and SPME-GC/MS Platforms
Source: Molecules. 2020 Dec 5;25(23):5749. doi: 10.3390/molecules25235749 (PMC7730677; doi:10.3390/molecules25235749)
Supplement: Supplementary file 1 [file molecules-25-05749-s001.pdf]

molecules

MDPI

Article

Detection of Fungi and Oomycetes by Volatiles Using E-Nose And SPME-GC/MS Platforms

Jérémie Loulier [1], François Lefort [1,*], Marcin Stocki [2], Monika Asztemborska [3], Rafał Szmigielski [3], Krzysztof Siwek [4], Tomasz Grzywacz [4], Tom Hsiang [5], Sławomir Ślusarski [6], Tomasz Oszako [2,6], Marcin Klisz [7], Rafał Tarakowski [8] and Justyna Anna Nowakowska [9,*]

[1] InTNE (Plants & Pathogens Group), hepia, University of Applied Sciences and Arts of Western Switzerland, 150 route de Presinge, 1254 Jussy, Switzerland; jeremie.loulier@bluewin.ch

[2] Institute of Forest Sciences, Faculty of Civil Engineering and Environmental Sciences, Bialystok University of Technology, Wiejska 45E, 15–351 Bialystok, Poland; m.stocki@pb.edu.pl (M.S.); T.Oszako@ibles.waw.pl (T.O.)

[3] Institute of Physical Chemistry, Polish Academy of Sciences, Kasprzaka 44/52, 01–224 Warsaw, Poland; monika@ichf.edu.pl (M.A.); ralf@ichf.edu.pl (R.S.)

[4] Faculty of Electrical Engineering, Warsaw University of Technology, Koszykowa 75, 00–661 Warsaw, Poland; krzysztof.siwek@ee.pw.edu.pl (K.S.); tomasz.grzywacz@ee.pw.edu.pl (T.G.)

[5] Environmental Sciences, University of Guelph, Guelph, Ontario N1G 2W1, Canada; thsiang@uoguelph.ca

[6] Forest Protection Department, Forest Research Institute, Braci Leśnej 3, 05–090 Sękocin Stary, Poland; S.Slusarski@ibles.waw.pl

[7] Department of Silviculture and Genetics, Forest Research Institute, Braci Leśnej 3, 05–090 Sękocin Stary, Poland; M.Klisz@ibles.waw.pl

[8] Faculty of Physics, Warsaw University of Technology, Koszykowa 75, 00–662 Warsaw, Poland; Rafal.Tarakowski@pw.edu.pl

[9] Institute of Biological Sciences, Cardinal Stefan Wyszynski University in Warsaw, Wóycickiego 1/3 Street, 01–938 Warsaw, Poland

* Correspondence: francois.lefort@hesge.ch (F.L.); j.nowakowska@uksw.edu.pl (J.A.N.); Tel.: +41-22-546-68-27 (F.L.); Tel. +48-22-569-6838 (J.A.N.)

Supplementary files:

Table 1. Composition of the sensor arrays used for e-nose sample detection tests. 1-8: Tagoshima gas sensors were purchased from Figaro Engineering Inc. (Osaka, Japan); 9-10: Relative humidity and temperature sensors were manufactured by Honeywell (Morristowne, New Jersey, USA) and Texas Instruments (Dallas, Texas, USA), respectively.

Sensor	Model	Sensitivity Range
1	TGS2600-B00 [1)]	Air contaminants such as hydrogen, ethanol, carbon monoxide, etc.
2	TGS2610-C00	Smaller than TGS2610-D00, this sensor quickly reacts to liquefied petroleum gases (LPG) like propane, butane, etc.
3	TGS2610-D00	Bigger than TGS2610-C00, this sensor includes a filter material preventing gases such as ethanol to interfere with the detection process, thus increasing its selectivity towards LPG (propane, butane, etc.).
4	TGS2611-C00	Smaller than TGS2611-E00, this sensor quickly reacts to methane.
5	TGS2611-E00	Bigger than TGS2611-C00, this sensor includes a filter material preventing gases such as ethanol to interfere with the detection process, thus increasing its selectivity towards methane.
6	TGS2612-D00 [1)]	Methane, propane, iso-butane

7	TGS2620-C00 [1)]	Alcohol, solvent vapors
8	TGS2602-C00 [1)]	Ammonia and hydrogen sulphide
9	HIH-3610-002	Relative humidity sensor consisting of a trimmed thermoset polymer capacitive sensing element with on-chip integrated signal conditioning
10	LM35DH	Precision integrated-circuit temperature device with an output voltage linearly proportional to Centigrade temperature

[1)] sensors for detection of relative humidity and ambient temperature

Table 2. Pearson's correlation coefficients between variables and the first two major components, and contribution of variables in principal components and variation explained.

Variables	Pearson's correlation		Contribution in PCs [%]	
	PC1	PC2	PC1	PC2
SEN_1	0.99	-0.03	8.78	0.19
SEN_2	1.00	-0.03	8.81	0.19
SEN_3	0.97	-0.17	8.38	7.23
SEN_4	0.92	-0.31	7.51	24.81
SEN_5	0.99	-0.04	8.69	0.48
SEN_6	0.98	0.00	8.58	0.00
SEN_7	0.97	-0.01	8.32	0.03
SEN_8	0.84	0.49	6.23	61.76
SEN_9	0.98	0.1	8.56	2.74
SEN_10	0.99	0.09	8.63	2.25
SEN_11	0.99	-0.02	8.77	0.12
SEN_12	0.99	-0.03	8.74	0.21
Variance explained			93.87	03.26

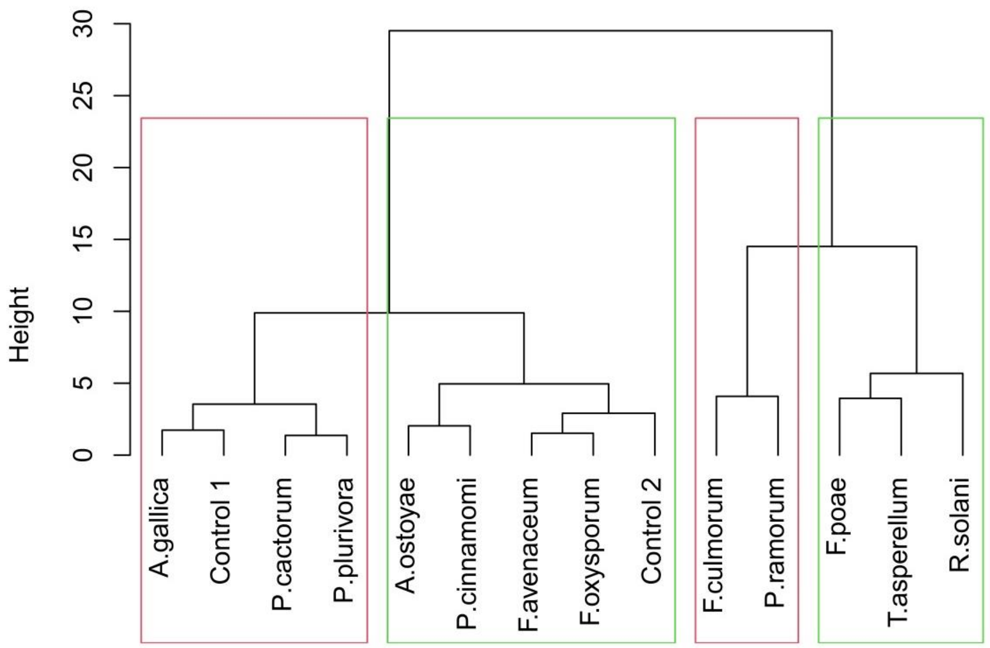

Figure 1. Hierarchical clustering of the microorganism-induced sensor signals based on Euclidean distance and Ward's minimum variance clustering method. The groups of similarities were clustered. Control 1 - Empty flask; Control 2 - PDA medium.
